# An ecological study on the association between International Health Regulations (IHR) core capacity scores and the Universal Health Coverage (UHC) service coverage index

**DOI:** 10.1186/s12992-022-00808-6

**Published:** 2022-02-08

**Authors:** Yuri Lee, Siwoo Kim, Jungju Oh, Sieun Lee

**Affiliations:** 1 Department of Health and Medical Information, Myongji College, Seoul, Republic of Korea; 2 Asian Institute for Bioethics and Health Law (WHO Collaborating Centre for Health Law and Bioethics), Yonsei University, Seoul, Republic of Korea

**Keywords:** International Health Regulations (2005), Joint External Evaluation, States Parties Self-Assessment Annual Reporting, Universal Health Coverage

## Abstract

**Background:**

The pandemic situation due to COVID-19 highlighted the importance of global health security preparedness and response. Since the revision of the International Health Regulations (IHR) in 2005, Joint External Evaluation (JEE) and States Parties Self-Assessment Annual Reporting (SPAR) have been adopted to track the IHR implementation stage in each country. While national IHR core capacities support the concept of Universal Health Coverage (UHC), there have been limited studies verifying the relationship between the two concepts. This study aimed to investigate empirically the association between IHR core capacity scores and the UHC service coverage index.

**Method:**

JEE score, SPAR score and UHC service coverage index data from 96 countries were collected and analyzed using an ecological study design. The independent variable was IHR core capacity scores, measured by JEE 2016-2019 and SPAR 2019 from the World Health Organization (WHO) and the dependent variable, UHC service coverage index, was extracted from the 2019 UHC monitoring report. For examining the association between IHR core capacities and the UHC service coverage index, Spearman’s correlation analysis was used. The correlation between IHR core capacities and UHC index was demonstrated using a scatter plot between JEE score and UHC service coverage index, and the SPAR score and UHC service coverage index were also presented.

**Result:**

While the correlation value between JEE and SPAR was 0.92 (*p* < 0.001), the countries’ external evaluation scores were lower than their self-evaluation scores. Some areas such as available human resources and points of entry were mismatched between JEE and SPAR. JEE was associated with the UHC score (*r* = 0.85, *p* < 0.001) and SPAR was also associated with the UHC service coverage index (*r* = 0.81, *p* < 0.001). The JEE and SPAR scores showed a significant positive correlation with the UHC service coverage index after adjusting for several confounding variables.

**Conclusion:**

The study result supports the premise that strengthening national health security capacities would in turn contribute to the achievement of UHC. With the help of the empirical result, it would further guide each country for better implementation of IHR.

## Introduction

Recent threats to global health security, including influenza A H1N1 (2009), Ebola virus disease (2014), MERS-Cov (2015), Zika virus disease (2016), and COVID-19 (2019), have emphasized the importance of strengthening global health security capacities more than ever. The International Health Regulations (IHR) 2005 provide an overarching legal framework designed to help countries build prevention, detection, and response capacities to deal with public health risks. The IHR has committed World Health Organization (WHO) member states to participate in international surveillance networks by reviewing and implementing sound surveillance strategies that contribute to global outbreak intelligence [1].

As 196 countries, including 194 WHO member states, agreed to report the implementation of the IHR to the World Health Assembly (Resolution WHA 58.3) [2], the IHR Monitoring and Evaluation Framework was then adopted to report their progress in implementing IHR. The two key features of the IHR Monitoring and Evaluation Framework are mandatory States Parties Self-Assessment Annual Reporting (SPAR) and voluntary Joint External Evaluation (JEE) [3]. The SPAR tool consists of 13 capacities and is an annual self-assessment tool, while the JEE tool evaluates 19 technical areas and is recommended to be done every 4-5 years. Despite these differences, both SPAR and JEE have similar structures and are complementary to each other for evaluating IHR core capacities [3, 4].

Understanding the mutual relationship to reinforce global health security capacities and UHC is a recent, yet crucial concept for creating synergistic effects [1, 5]. Previous studies have attempted to analyze whether the technical components of the IHR core capacity scores converge to each other, whether they contribute to IHR implementation, and whether their evaluation reflects the actual global health security capacity level [6–9]. Previous research also reports lessons learned for IHR implementation and the possible mechanisms for IHR implementation [10, 11]. One study comparing global health security and UHC showed a significant relationship between the two indices [12]. However, there have been limited and controversial studies on the relationship between IHR core competencies and UHC.

Therefore, this study first aims to examine the global distribution and association between the JEE score and SPAR score to determine whether one could represent the other. Second, the study examines the differences between IHR core capacity scores (JEE score and SPAR score) and the UHC service coverage index. Third, the study analyzes the association between IHR core capacity scores and the UHC service coverage index to provide empirical evidence on the two global health agendas.

## Methods

### Study design

**Fig. 1:**
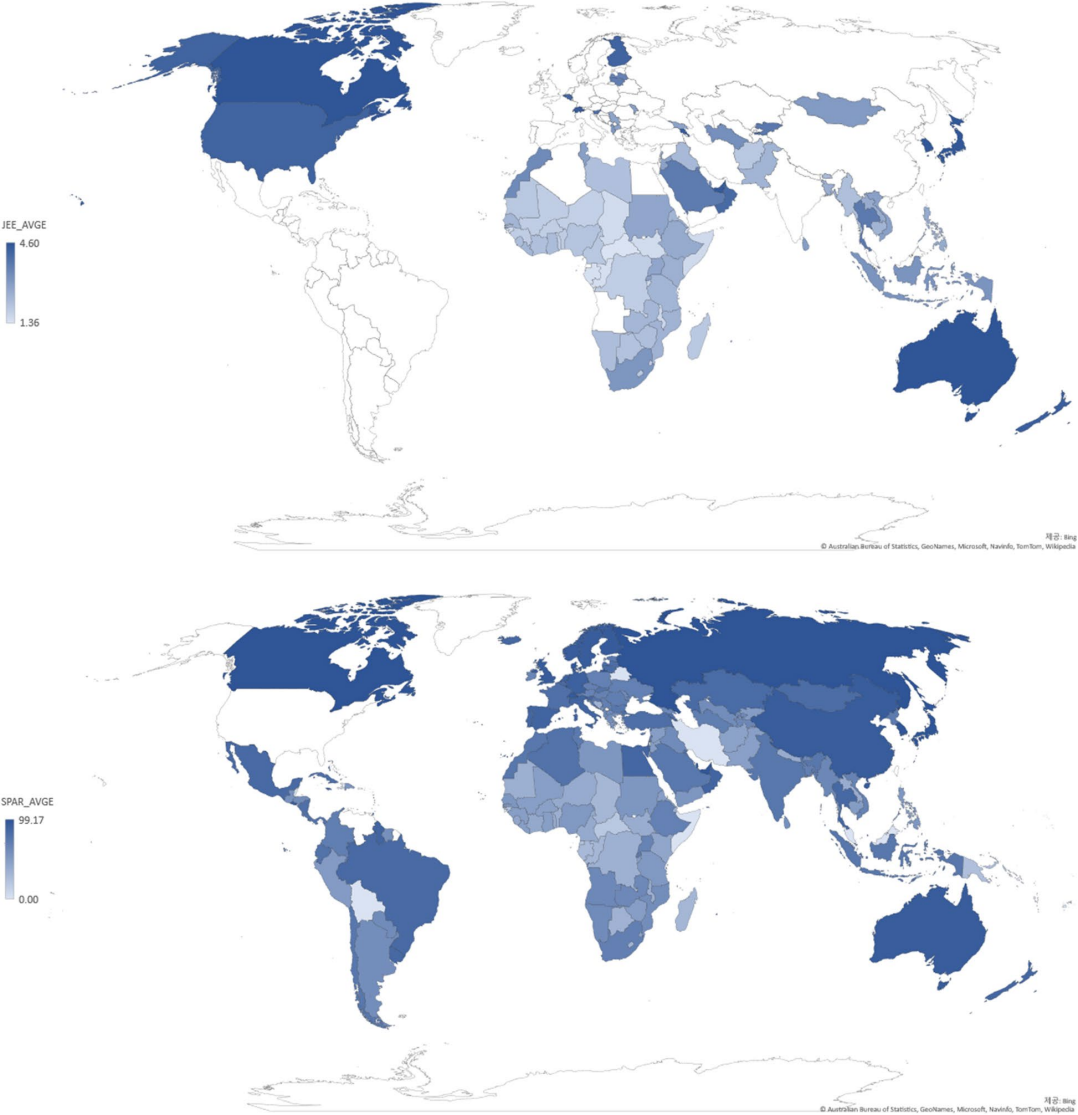
Global Distribution of JEE and SPAR scores

An ecological study design was used in this study to investigate the correlation between IHR core capacity scores and the UHC service coverage index (Fig. 1). The study is based on analyses of data collected from 96 countries for JEE, SPAR, and the UHC service coverage index. The IHR core capacity scores, which were measured by JEE 2016-2019 and SPAR 2019 from the World Health Organization (WHO), were used as independent variables. The dependent variable, i.e., the UHC service coverage index, was extracted from the 2019 UHC monitoring report.

This study identified the confounding variables that may affect UHC service coverage. This study selected the confounding variables that may affect UHC service coverage. UHC supports the idea that health service is available to everyone without causing financial hardship. Tracking the progress towards UHC uses two specific indicators, health services coverage and financial risk protection coverage. Previous studies on UHC identified that socio-demographic index, government health expenditure and governance show positive association with UHC service coverage. The selection principle of confounding variables is related to both core explanatory variables and dependent variables. Thus, the confounding variables in this study include GDP per-capita, current health expenditure, infant mortality rate, life expectancy at birth, hospital beds, medical doctors, nursing and midwifery personnel, population ages under five, population ages 65 and above [9, 10].

Four models were demonstrated to understand the factors of JEE and other independent variables affecting UHC service coverage, and SPAR and other independent variables affecting UHC service coverage. Model 1 includes the global health security index of either JEE or SPAR overall mean score and population variables (population age under five, population age 65 and above) as independent variables, Model 2 incorporates the variables used in Model 1 and economic variables (GDP per capita, current health expenditure), model 3 includes the variables used in model 2 and variables related to medical resources (hospital beds, medical doctors, nursing, and midwifery personnel), and Model 4 includes the variables used in Model 3 and variables related to health status (infant mortality rate, life expectancy at birth).

### Country data

JEE 2016-2019, SPAR 2019, and UHC service coverage index 2017 data from 1st March, 2021 to 31st March, 2021 were extracted [13–15]. Online databases from the World Bank, WHO Global Health Observatory, and United Nations provided GDP per capita, current health expenditure, infant mortality rate, life expectancy at birth, hospital beds, medical doctors, nursing and midwifery personnel, population age under five, population age 65 and above. GDP per capita, current health expenditure, and infant mortality rates were available from the World Bank [16–18]. Data on life expectancy at birth, hospital beds, medical doctors, and nursing and midwifery personnel were available from the WHO Global Health Observatory [19–22]. The United Nations website was the other source of data for population age under five and population age 65 and above [23, 24] (Table 1).

**Table 1:** Data sources and definitions

Variable	Source	Definition	Time period
JEE	WHO	Joint External Evaluation	2016-2019
SPAR	WHO	States Parties Self-Assessment Annual Reporting	2019
UHC Service Coverage	WHO	Universal Health Coverage	2020
GDP per-capita	World Bank	GDP per-capita	2019
Current health expenditure	World Bank	Current health expenditure (% of GDP)	2018
Infant mortality rate	World Bank	Infant mortality rate (deaths per 1000 live births)	2019
Life expectancy at birth	WHO Global Health Observatory	Life expectancy at birth (years)	2020
Hospital beds	WHO Global Health Observatory	Hospital beds (per 10,000 population)	2020
Medical doctors	WHO Global Health Observatory	Medical doctors (per 10,000 population)	2021
Nursing and midwifery personnel	WHO Global Health Observatory	Nursing and midwifery personnel (per 10,000 population)	2021
Population ages under 5	United Nations	Population ages under 5 (% of total population)	2019
Population ages 65 and above	United Nations	Population ages 65 and above (% of total population)	2019

The JEE tool contains 19 technical areas, represented by 48 indicators. Each technical area represents the mean scores of the indicators. The indicator’s scoring system is based on a five-point ordinal scale from 1 to 5, reflecting higher capacity as the score increases. The SPAR tool consists of 24 indicators from the 13 IHR capacities needed to detect, assess, notify, and respond to public health events of national and international concern. The level of SPAR is expressed as the average of all indicators, which are calculated as a percentage of performance based on a scale of 1 to 5. Recognizing the conceptual similarities between JEE and SPAR, the technical areas were matched and categorized into 15 areas. The overall mean values of JEE and SPAR were retrieved and used for the statistical analysis, except for the spider diagram comparing JEE and SPAR, where the value of JEE was multiplied by 20 to ensure that both JEE and SPAR could have the same scale.

### Statistical Analysis

Statistical analyses were performed using SPSS version 25.0. A spider diagram was used to visually explain the relationship between the JEE and SPAR scores. The correlation analysis between JEE and SPAR further supported this relationship. Furthermore, Pearson correlation analysis was used to test the association between the IHR core capacity scores and the UHC service coverage index. Descriptive analysis and one-way analysis of variance (ANOVA) were used to describe the general characteristics of the selected countries and compare the JEE scores, SPAR scores, and the UHC service coverage index by population, economic index, human resources for health, and health indicators of countries. The scatterplots between the JEE score and the UHC service coverage index, as well as the SPAR score and the UHC service coverage index, were presented to support this relationship. Lastly, multiple regression analysis was used to test the effect of the IHR core capacity scores on the UHC service coverage index. We used a variance inflation factor (VIF) to confirm that multicollinearity did not occur between the explanatory variables (VIF < 10). The level of statistical significance was set at *p* < 0.05.

## Results

**Fig. 2:**
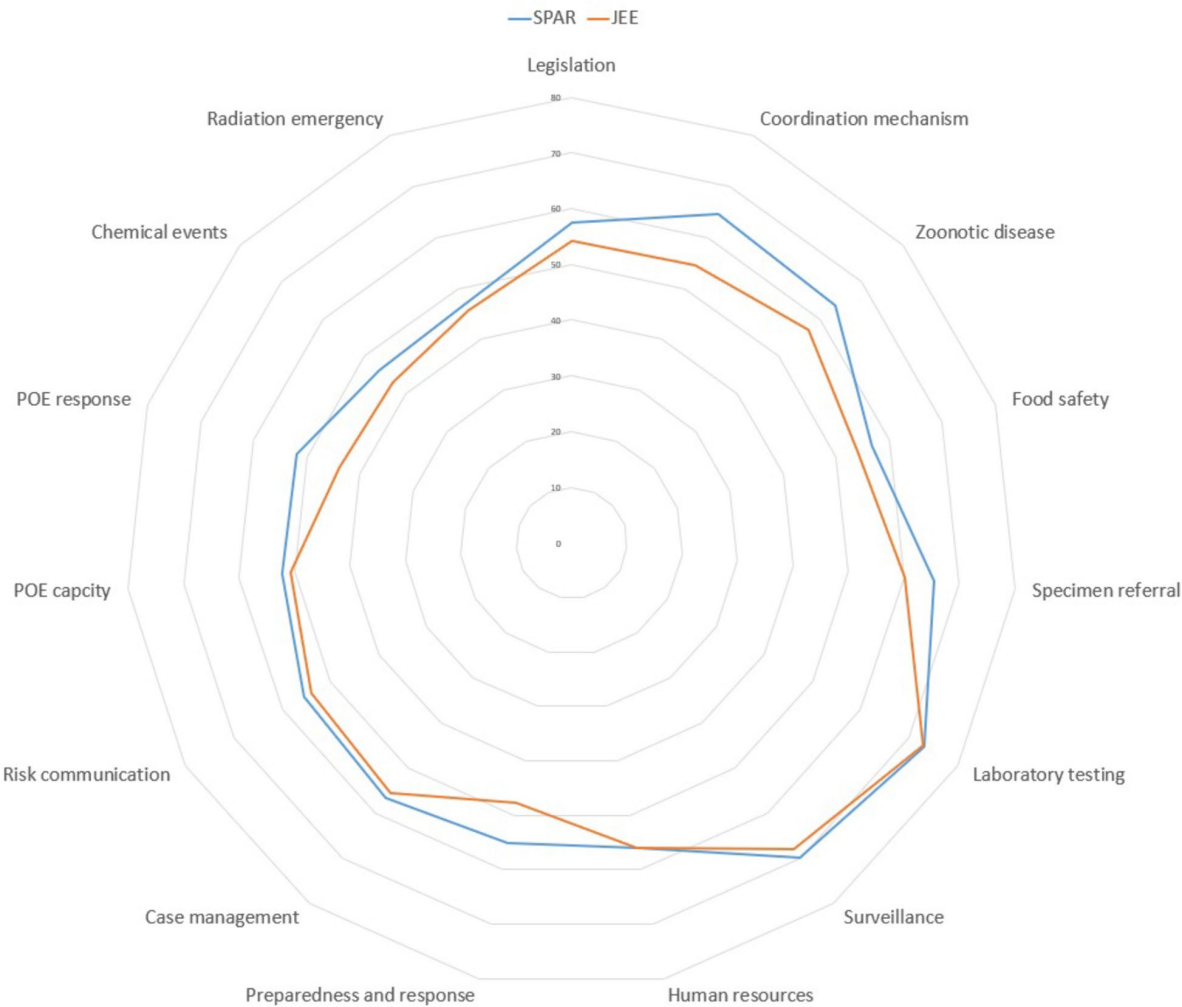
Spider Diagram of JEE and SPAR

The global distribution of JEE and SPAR scores (Fig. 1) shows similar patterns between the JEE and SPAR scores. SPAR and JEE scores were compared by matching the variables of the two assessment tools based on their contextual similarities. With the lowest *r*-value as 0.514 and the highest *r*-value as 0.786, a significant relationship between JEE and SPAR was identified (Table 2). While the response and radiation areas converged in the spider diagram, overall, the JEE score was more conservative than the SPAR score (Fig. 2).

**Table 2:** Connecting variables between SPAR and JEE

Category	SPAR	JEE	SPAR	JEE	SPCC
			Mean ± SD	Mean ± SD	*r*
Prevention	Legislation, laws, regulations, policy, administrative requirements or other government instruments to implement the IHR (2005)	Legislation, laws, regulations, administrative requirements, policies or other government instruments in a place are sufficient for implementation of IHR (2005)	57.4 ± 26.1	2.71 ± 1.26	0.758***
	Multi-sectoral IHR coordination mechanisms	A functional mechanism is established for the coordination and integration of relevant sectors in the implementation of IHR	64.7 ± 24.9	2.73 ± 1.27	0.672***
	Collaborative effort on activities to address zoonosis	Surveillance systems in place for priority zoonotic diseases/pathogens	63.6 ± 26.0	2.86 ± 1.02	0.599***
		Veterinary or animal health workforce			
		Mechanisms for responding to infectious and potential zoonotic diseases are established and functional			
	Multisectoral collaboration mechanism for food safety events	Mechanisms for multisectoral collaboration are established to ensure rapid response to food safety emergencies and outbreaks of foodborne diseases	56.8 ± 28.5	2.70 ± 1.31	0.700***
Detection	Specimen referral and transport system	Specimen referral and transport system	65.5 ± 22.2	3.07 ± 1.28	0.756***
	Access to laboratory testing capacity for priority diseases	Laboratory testing for detection of priority diseases	73.2 ± 23.6	3.64 ± 1.03	0.616***
	Early warning function: indicator- and event-based surveillance	Indicator- and event-based surveillance systems	69.8 ± 18.7	3.40 ± 0.84	0.661***
	Human resources to implement IHR (2005) capacities	Human resources available to implement IHR core capacity requirements	55.9 ± 25.7	2.80 ± 1.04	0.514***
Response	Planning for emergency preparedness and response mechanism	National multi-hazard public health emergency preparedness and response plan is developed and implemented	55.1 ± 25.6	2.39 ± 1.43	0.784***
	Case management capacity for IHR relevant hazards	Case management procedures implemented for IHR relevant hazards	56.7 ± 25.4	2.77 ± 1.23	0.666***
	Capacity for emergency risk communications	Risk communication systems (plans, mechanisms, etc.)	55.4 ± 27.7	2.70 ± 0.85	0.715***
		Internal and partner communication and coordination			
		Public communication			
		Communication engagement with affected communities			
		Dynamic listening and rumour management			
Other criteria	Core capacity requirements at all times for designated airports, ports and ground crossings	Routine capacities established at points of entry	52.2 ± 28.2	2.57 ± 1.36	0.678***
	Effective public health response at points of entry	Effective public health response at points of entry	51.8 ± 28.4	2.19 ± 1.39	0.771***
	Resources for detection and alert	Mechanisms established and functioning for detecting and responding to chemical events or emergencies	46.5 ± 30.5	2.16 ± 1.27	0.774***
	Capacity and resources	Mechanisms established and functioning for detecting and responding to radiological and nuclear emergencies	46.9 ± 31.0	2.28 ± 1.28	0.786***

*p < 0.05, **p < 0.01, ***p < 0.001

The 96 countries were relatively evenly distributed by GDP per capita for high income (21.9%), upper-middle income (20.8%), lower-middle income (33.3%), and low income (22.9%) groups. Twenty-three (24%) of the analyzed countries showed less than 4% of health expenditure in GDP, and 51 (53.1%) showed a health expenditure percentage between 4.01 and 8% (Table 3).

**Table 3:** Differences of JEE, SPAR, UHC by independent variables (*n* = 96)

Variables	Categories	N (%)	JEE		SPAR		UHC Service Coverage	
			Mean ± SD	F(*p*)	Mean ± SD	F(*p*)	Mean ± SD	F(*p*)
GDP per-capita	High income	21 (21.9)	44.02 ± 0.55	60.43***	86.54 ± 12.29	46.63***	78.43 ± 7.08	77.67***
	Upper-middle income	20 (20.8%)	2.80 ± 0.64		62.94 ± 14.42		64.45 ± 7.90	
	Lower-middle income	32 (33.3%)	2.25 ± 0.53		47.59 ± 13.13		51.44 ± 11.43	
	Low-income	22 (22.9%)	2.03 ± 0.45		44.75 ± 13.40		40.05 ± 6.20	
Current health expenditure (% of GDP)	0-4	23 (24.0%)	2.32 ± 0.62	3.05**	53.08 ± 18.82	1.98	49.39 ± 11.25	3.11**
	4.01-8	51 (53.1%)	2.70 ± 0.84		57.67 ± 19.07		59.08 ± 15.10	
	8.01-12	17 (17.7%)	3.27 ± 1.20		69.98 ± 24.93		65.82 ± 19.16	
	12.01-16	1 (1.0%)	2.83 ± 0.00		46.00 ± 0.00		47.00 ± 0.00	
	16.01-20	2 (2.1%)	3.24 ± 1.46		49.17 ± 37.71		61.50 ± 31.82	
Infant mortality rate (death per 1000 live births)	0-20	39 (40.6%)	3.57 ± 0.73	38.25***	77.37 ± 15.28	32.51***	73.59 ± 8.45	69.34***
	20.01-40	30 (31.3%)	2.27 ± 0.47		50.00 ± 13.50		51.13 ± 9.53	
	40.01-60	16 (16.7%)	2.00 ± 0.34		42.14 ± 10.58		43.50 ± 4.90	
	60.01-80	8 (8.3%)	1.83 ± 0.29		40.43 ± 8.28		36.38 ± 7.78	
	80.01-100	2 (2.1%)	1.79 ± 0.60		30.84 ± 16.50		36.00 ± 4.24	
Life expectancy at birth (years)	40-60	5 (5.2%)	1.87 ± 0.37	28.66***	14.79 ± 12.98	21.10***	42.00 ± 15.64	44.19***
	60.01-70	44 (45.8)	2.16 ± 0.46		46.75 ± 13.28		46.05 ± 8.70	
	70.01-80	34 (35.4%)	3.12 ± 0.70		68.93 ± 15.37		68.12 ± 7.75	
	80.01-100	12 (12.5%)	3.88 ± 1.19		80.69 ± 26.93		75.92 ± 19.06	
Hospital beds (per 10,000 population)	0-30	70 (72.9%)	2.49 ± 0.84	8.39***	54.65 ± 20.14	6.82***	53.30 ± 15.47	10.56***
	30.01-60	19 (19.8%)	3.30 ± 0.84		69.47 ± 16.47		71.05 ± 8.58	
	60.01-90	2 (2.1%)	3.32 ± 0.45		82.92 ± 0.59		67.50 ± 7.78	
	90.01-120	0(%)	0.00 ± 0.00		0.00 ± 0.00		0.00 ± 0.00	
	120.1-150	2 (2.1%)	4.53 ± 0.01		97.92 ± 0.59		84.50 ± 2.12	
Medical doctors (per 10,000 population)	0-20	64 (66.7%)	2.28 ± 0.61	33.20***	49.72 ± 15.77	23.92***	49.66 ± 12.11	36.41***
	20.01-40	24 (25.0%)	3.59 ± 0.77		77.81 ± 17.01		75.04 ± 8.35	
	40.01-60	5 (5.2%)	4.19 ± 0.33		87.33 ± 6.71		77.40 ± 6.43	
	60.01-80	1 (1.0%)	2.82 ± 0.00		62.50 ± 0.00		66.00 ± 0.00	
Nursing and midwifery personnel (per 10,000 population)	0-50	70 (72.9%)	2.43 ± 0.74	17.90***	53.18 ± 17.88	12.67***	52.19 ± 14.08	18.72***
	50.01-100	17 (17.7%)	3.31 ± 0.82		70.79 ± 19.62		70.41 ± 8.35	
	100.01-150	4 (4.2%)	4.47 ± 0.19		93.54 ± 7.28		85.75 ± 2.75	
	150.01-200	2 (2.1%)	4.36 ± 0.12		95.42 ± 0.59		83.50 ± 0.71	
Population ages under 5 (% of total population)	0-5	16 (16.7%)	3.86 ± 0.70	37.94***	85.52 ± 13.85	42.49***	77.13 ± 8.03	59.27***
	5.01-10	27 (28.1)	3.14 ± 0.79		69.12 ± 14.51		67.85 ± 10.61	
	10.01-15	33 (34.4%)	2.22 ± 0.55		44.96 ± 14.17		49.03 ± 10.97	
	15.01-20	19 (19.8%)	1.97 ± 0.36		45.43 ± 10.99		41.00 ± 6.44	
	20.01-30	0 (0%)	0.00 ± 0.00		0.00 ± 0.00		0.00 ± 0.00	
Population ages 65 and above (% of total population)	0-7	68 (70.8%)	2.33 ± 0.70	26.54***	50.73 ± 17.37	22.24***	50.35 ± 12.79	30.15***
	7.01-14	12 (12.5%)	3.25 ± 0.67		70.82 ± 11.60		72.08 ± 6.11	
	14.01-21	13 (13.5%)	3.91 ± 0.67		84.23 ± 12.77		77.92 ± 9.97	
	21.01-50	2 (2.1%)	4.40 ± 0.21		96.25 ± 2.94		80.50 ± 3.54	

*p < 0.05, **p < 0.01, ***p < 0.001

Table 3 shows multiple disparities in IHR core capacity scores and the UHC service coverage index. There was a significant difference (*p* < 0.05) between JEE, SPAR, and UHC service coverage in all groups except current health expenditure, i.e., GDP per capita, infant mortality rate, life expectancy at birth, hospital beds, medical doctors, nursing and midwifery personnel, population age under 5, and population age 65 and above. The JEE and UHC service coverage were significant in current health expenditure, while SPAR was not significant. In JEE and UHC service coverage, the F statistic was the highest in the GDP per-capita (60.43 and 77.67) and the lowest in the current health expenditure (3.05 and 3.11), respectively. In SPAR, the highest F statistic was GDP per capita (46.63), and the lowest was hospital beds (6.82).

**Fig. 3:**
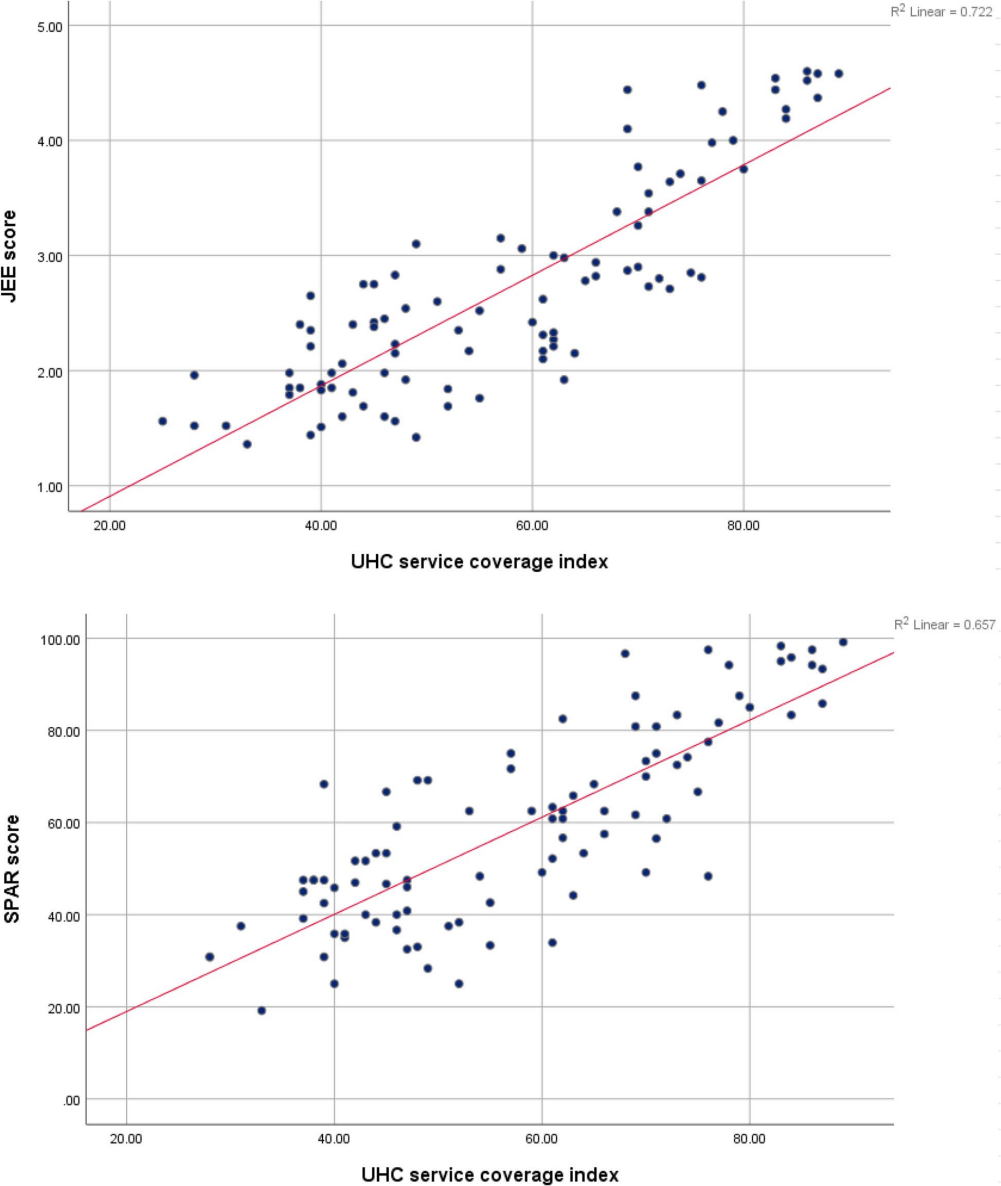
Scatter plots of JEE and SPAR in relation to UHC

The scatter plots of JEE and SPAR scores in relation to the UHC service coverage index were positively associated with the UHC service coverage index (Fig. 3). Multiple regression analysis was then performed using the four models. JEE affected UHC service coverage in all four models, while the effect of SPAR was valid in only three models. JEE scores, together with population, economic resources, health resources, and health status variables, affected the UHC service coverage index. The variables in Model 4 could explain 85.9% (adjusted *R*^2^ = 0.859, *β* = 0.275, *p* < 0.001) of the variance in UHC service coverage when JEE was taken as an independent variable. It showed that SPAR, together with population, economic resources, and health resources, affects UHC service coverage. The variables in Model 3 could explain 82.3% (adjusted *R*^2^ = 0.823, *β* = 0.240, *p* < 0.001) of the variance in UHC service coverage when SPAR was taken as an independent variable. However, SPAR was not valid in Model 4 (Table 4).

**Table 4:** Multiple Regression Analysis of IHR core capacity scores and UHC service coverage Index

Independent variable	Model		Β (SE)	*β*	t(*p*)	F(*p*)	^*adj*^*R*^2^	VIF
JEE	1	(Constant)	60.374 (6.222)		9.703***	153.075***	0.829	
		JEE score	0.422 (1.263)	0.422	5.909***			2.801
		Population ages under 5	−2.044 (0.290)	−0.556	−7.061***			3.417
		Population ages 65 and above	− 0.047 (0.181)	− 0.017	− 0.259			2.394
	2	(Constant)	61.225 (6.319)		9.688***	89.152***	0.826	
		JEE score	6.475 (1.449)	0.371	4.468***			3.674
		Population ages under 5	−1.997 (0.296)	−0.548	−6.747***			3.527
		Population ages 65 and above	−0.086 (0.192)	− 0.032	− 0.446			2.731
		GDP per-capita	0.000 (0.000)	0.079	1.162			2.455
		Current health expenditure	0.149 (0.276)	0.027	0.540			1.289
	3	(Constant)	59.399 (6.215)		9.558***	59.481***	0.839	
		JEE score	6.064 (1.437)	0.352	4.219***			3.874
		Population ages under 5	−1.857 (0.303)	−0.514	−6.132***			3.916
		Population ages 65 and above	−0.370 (0.213)	− 0.139	− 1.732			3.599
		GDP per-capita	0.000 (0.000)	0.043	0.534			3.614
		Current health expenditure	0.228 (0.287)	0.040	0.796			1.438
		Hospital beds	0.076 (0.045)	0.109	1.703			2.297
		Medical doctors	0.027 (0.076)	0.026	0.355			3.024
		Nursing and midwifery personnel	0.041 (0.035)	0.095	1.184			3.592
	4	(Constant)	66.626 (13.138)		5.300***	55.704***	0.859	
		JEE score	4.739 (1.393)	0.275	3.403**			4.153
		Population ages under 5	−1.317 (0.330)	−0.365	−3.996***			5.303
		Population ages 65 and above	−0.291 (0.201)	− 0.110	− 1.448			3.651
		GDP per-capita	0.000 (0.000)	0.069	0.887			3.850
		Current health expenditure	0.462 (0.276)	0.082	1.675			1.519
		Hospital beds	0.061 (0.042)	0.087	1.439			2.342
		Medical doctors	−0.016 (0.073)	−0.016	−0.227			3.113
		Nursing and midwifery personnel	0.026 (0.033)	0.060	0.785			3.680
		Infant mortality rate	− 0.234 (0.064)	− 0.313	− 3.637*			4.712
		Life expectancy at birth	− 0.094 (0.152)	− 0.044	− 0.620			3.219
SPAR	1	(Constant)	66.172 (6.366)		10.394***	128.339***	0.804	
		SPAR score	0.257 (0.056)	0.334	4.589***			2.512
		Population ages under 5	−2.166 (0.307)	−0.595	−7.064***			3.370
		Population ages 65 and above	0.079 (0.188)	0.029	0.418			2.338
	2	(Constant)	67.087 (6.380)		10.515***	80.299***	0.810	
		SPAR score	0.204 (0.062)	0.266	3.318**			3.146
		Population ages under 5	−2.153 (0.307)	−0.591	−7.018***			3.474
		Population ages 65 and above	−0.047 (0.200)	− 0.018	−0.236			2.718
		GDP per-capita	0.000 (0.000)	0.122	1.760			2.355
		Current health expenditure	0.269 (0.287)	0.048	0.936			1.284
	3	(Constant)	64.970 (6.352)		10.229***	53.349***	0.823	
		SPAR score	0.182 (0.061)	0.240	3.004**			3.253
		Population ages under 5	−1.967 (0.319)	−0.544	−6.161***			3.970
		Population ages 65 and above	−0.346 (0.223)	− 0.130	−1.547			3.599
		GDP per-capita	0.000 (0.000)	0.085	1.022			3.543
		Current health expenditure	0.292 (0.300)	0.052	0.975			1.434
		Hospital beds	0.078 (0.047)	0.112	1.666			2.301
		Medical doctors	0.055 (0.079)	0.053	0.689			2.974
		Nursing and midwifery personnel	0.043 (0.037)	0.099	1.170			3.614
	4	(Constant)	74.067 (14.133)		5.241***	50.154***	0.845	
		SPAR score	0.113 (0.060)	0.150	1.897			3.618
		Population ages under 5	−1.404 (0.346)	−0.388	−4.058***			5.329
		Population ages 65 and above	−0.271 (0.210)	−0.102	−1.289			3.646
		GDP per-capita	0.000 (0.000)	0.113	1.392			3.858
		Current health expenditure	0.528 (0.288)	0.093	1.834			1.507
		Hospital beds	0.062 (0.044)	0.089	1.402			2.346
		Medical doctors	0.006 (0.076)	0.005	0.073			3.076
		Nursing and midwifery personnel	0.025 (0.035)	0.058	0.723			3.726
		Infant mortality rate	−0.247 (0.069)	−0.330	−3.588**			4.919
		Life expectancy at birth	−0.068 (0.160)	− 0.032	−0.426			3.255

Model 1 – population indicator was adjusted

Model 2 – population and economic indicators were adjusted

Model 3 – population, economic and health resource indicators adjusted

Model 4 – population, economic, health resource, and health status indicators were adjusted

*p < 0.05, **p < 0.01, ***p < 0.001

## Discussion

This study showed a significant association between JEE and SPAR; at the same time, lower JEE scores compared to SPAR scores were identified. In addition, a strong association between IHR core capacities and UHC service coverage was demonstrated using empirical data. All four models using JEE as the independent variable were valid, while only three models that used SPAR as an independent variable were valid.

Several studies have reported similar findings in terms of the relationship between JEE and SPAR. While not completely converging, the indicators of JEE and SPAR show a high level of correlation when mapped together [3, 12, 25, 26]. Considering the complementary functions of JEE and SPAR, they are both relevant to each other and are representative of the IHR core capacity scores. As identified in the study, similarities and differences were found between JEE and SPAR in the spider diagram (Fig. 2). Indeed, gaps exist between the data due to errors in matching the contextual similarities. Although indicators of JEE and SPAR are presented with identical keywords, they are likely to assess different aspects of IHR core capacity. At the same time, it can also be argued that the gaps are not significant, as can be seen in Fig. 3, meaning that they can be neglected.

Only recently has it been brought to attention by the global public health community that global health security is an integral part of public health functions [1]. The global response to the earlier infectious disease outbreaks gave us a lesson that preparedness and response to public health threats require intra-sectoral approaches, including governance, incident management, public health, health care, logistics, sociocultural and community initiatives, and global response [27]. A systematic review that analyzed the link between the Ebola outbreak and health systems concluded that ensuring an adequate and efficient health workforce, a strong health system, adequate service delivery, health financing and management, leadership, and governance all affected the countries’ performance regarding the Ebola disease outbreak [28]. In short, the preparedness and response to public health crises requires strengthening health systems and inter-sectoral approaches, which in turn contribute to improving global health security and achieving UHC.

Since an ecological study was performed here, the collected data were useful to explore the association between JEE and SPAR, and the association between global health security and UHC [29]. However, there is no effective way of taking into account or adjusting for other factors that influence the outcome. As a result, an apparent correlation between JEE and SPAR, and global health security and UHC could be misleading. It should be noted that all factors cannot be adjusted in the ecological study because in the real world, all known and unknown factors affect the dependent variable. Therefore, future studies should analyze the effects of the health system on global health security and on UHC, and analyze whether the health system has moderating effects.

Other possible limitations include reporting bias, as the study results are based on the data reported by each country, and data have been retrieved from various institutions and websites. Detection bias might have also affected the results because exact mechanisms between global health security capacity and UHC were not identified in the study; there might be some variables affecting them. Time bias could be another source of limitation because the data were not collected at the same time. However, this study used the latest data to analyze the results, to minimize reporting and time bias; each result was carefully reviewed to keep the risk of detection bias low.

While this study is prone to reporting, detection, and time bias, it is still reliable enough to explain the relationship between JEE and SPAR, and between IHR core capacity scores and UHC, with the empirical data. The study results support the premise that the two global agendas, global health security and UHC, do not stand alone, but are mutually interconnected. Focusing on one agenda would lead to inappropriateness in providing health services to the population without financial hardship, and preparing and responding to global health risks. Previous research has shown a strong correlation between JEE, health outcomes, and the function of the health system. Therefore, embedding global health security into UHC is crucial; global health security needs to be integrated with the health system [8, 5, 25, 30–33]. In addition, the JEE and SPAR are complementary to each other, and it is important to ensure that they maintain a certain level of convergence.

## Conclusion

Achieving UHC requires regional, national, and international efforts in health, social, and cross-cutting areas. This research attempted to verify the correlation between global health security capacity and UHC using data from 96 countries regarding JEE score, SPAR score, and the UHC service coverage index. The results showed that JEE and SPAR are strongly associated with UHC. However, the specific mechanism of how preparedness, detection, and response criteria of JEE and SPAR affect UHC remains a topic that needs to be addressed.

There is inadequate global preparedness for health security, and no country or region is fully prepared for global health security. Ensuring global health security requires prevention, detection, and response to emergencies at the national, regional, and global levels. The aspiration for global health security will not be realized without UHC; hence, the tension between global health security and UHC should be transformed into synergistic planning, financing, and implementation, through a diagonal investment and service delivery approach, including differentiated, integrated, and community-led services. In doing so, the health system and policies should be strengthened to ensure the implementation of IHR and achieving UHC.

## Data Availability

The datasets supporting the conclusions of this article are available from the website of World Health Organization, World Bank, and United Nations. Detailed website addresses are given in the manuscript and references [13–24].

## Abbreviations

JEE: Joint External Evaluation
SPAR: Self-Assessment Annual Reporting
UHC: Universal Health Coverage
